# Integrated disease surveillance and response implementation in Liberia, findings from a data quality audit, 2017

**DOI:** 10.11604/pamj.supp.2019.33.2.17608

**Published:** 2019-05-31

**Authors:** Thomas Nagbe, Kwuakuan Yealue, Trokon Yeabah, Julius Monday Rude, Musoka Fallah, Laura Skrip, Chukwuemeka Agbo, Nuha Mouhamoud, Joseph Chukwudi Okeibunor, Roland Tuopileyi, Ambrose Talisuna, Ali Ahmed Yahaya, Soatiana Rajatonirina, Joseph Asamoah Frimpong, Mary Stephen, Esther Hamblion, Tolbert Nyenswah, Bernice Dahn, Alex Gasasira, Ibrahima Socé Fall

**Affiliations:** 1 National Public Health Institute, Monrovia, Liberia; 2 World Health Organization, Monrovia, Liberia; 3 World Health Organization, Regional Office for Africa, Brazzaville, Congo; 4 Africa Field Epidemiology Network, Monrovia, Liberia

**Keywords:** Data quality assessment, disease surveillance information system, simple random sample, data accuracy, reliability and credibility, multi-stage cluster sampling, completeness, and timeliness integrated disease surveillance and response, health management information system (HMIS)/district health informative system two (DHIS2) database, case investigation forms and eDEWS

## Abstract

**Introduction:**

in spite of the efforts and resources committed by the division of infectious disease and epidemiology (DIDE) of the national public health institute of Liberia (NPHIL)/Ministry of health to strengthening integrated disease surveillance and response (IDSR) across the country, quality data management system remains a challenge to the Liberia NPHIL/MoH (Ministry of health), with incomplete and inconsistent data constantly being reported at different levels of the surveillance system. As part of the monitoring and evaluation strategy for IDSR continuous improvement, data quality assessment (DQA) of the IDSR system to identify successes and gaps in the disease surveillance information system (DSIS) with the aim of ensuring data accuracy, reliability and credibility of generated data at all levels of the health system; and to inform an operational plan to address data quality needs for IDSR activities is required.

**Methods:**

multi-stage cluster sampling that included **stage 1:** simple random sample (SRS) of five counties, **stage 2:** simple random sample of two districts and **stage 3:** simple random sample of three health facilities was employed during the study pilot assessment done in Montserrado County with Liberia institute of bio medical research (LIBR) inclusive. A total of thirty (30) facilities was targeted, twenty nine (29) of the facilities were successfully audited: one hospital, two health centers, twenty clinics and respondents included: health facility surveillance focal persons (HFSFP), zonal surveillance officers (ZSOs), district surveillance officers (DSOs) and County surveillance officers (CSOs).

**Results:**

the assessment revealed that data use is limited to risk communication and sensitization, no examples of use of data for prioritization or decision making at the subnational level. The findings indicated the following: 23% (7/29) of health facilities having dedicated phone for reporting, 20% (6/29) reported no cell phone network, 17% (5/29) reported daily access to internet, 56.6% (17/29) reported a consistent supply of electricity, and no facility reported access to functional laptop. It was also established that 40% of health facilities have experienced a stock out of laboratory specimens packaging supplies in the past year. About half of the surveyed health facilities delivered specimens through riders and were assisted by the DSOs. There was a large variety in the reported packaging process, with many staff unable to give clear processes. The findings during the exercise also indicated that 91% of health facility staff were mentored on data quality check and data management including the importance of the timeliness and completeness of reporting through supportive supervision and mentorship; 65% of the health facility assessed received supervision on IDSR core performance indicator; and 58% of the health facility officer in charge gave feedback to the community level.

**Conclusion:**

public health is a data-intensive field which needs high-quality data and authoritative information to support public health assessment, decision-making and to assure the health of communities. Data quality assessment is important for public health. In this review completeness, accuracy, and timeliness were the three most-assessed attributes. Quantitative data quality assessment primarily used descriptive surveys and data audits, while qualitative data quality assessment methods include primarily interviews, questionnaires administration, documentation reviews and field observations. We found that data-use and data-process have not been given adequate attention, although they were equally important factors which determine the quality of data. Other limitations of the previous studies were inconsistency in the definition of the attributes of data quality, failure to address data users’ concerns and a lack of triangulation of mixed methods for data quality assessment. The reliability and validity of the data quality assessment were rarely reported. These gaps suggest that in the future, data quality assessment for public health needs to consider equally the three dimensions of data quality, data use and data process. Measuring the perceptions of end users or consumers towards data quality will enrich our understanding of data quality issues. Data use is limited to risk communication and sensitization, no examples of use of data for prioritization or decision making at the sub national level.

## Introduction

Liberia and other countries in the WHO African Region continue to be affected by inconsistent data at all levels of the surveillance system, which may affect the implementation of the IDSR in a negative manner. Public health data are used to monitor trends in the health and wellbeing of the community and of health determinants. Also, they are used to assess the risks of adverse health effects associated with certain determinants, and the positive effects associated with protective factors. The data informs the development of public health policy and the establishment of priorities for investment in interventions aimed at modifying health determinants. They are also used to monitor and evaluate the implementation, cost and outcomes of public health interventions, and to implement surveillance of emerging health issues [1]. Thus, public health data can help public health agencies to make appropriate decisions, take effective and efficient action, and evaluate the outcomes [1, 2]. For example, health indicators set up the goals for the relevant government-funded public health agencies [3]. Data verification was conducted at selected sites by reviewing data sources that include but not limited to, Supportive Supervision Reports, Outbreak Reports, Maternal and Neonatal Death Surveillance and Response Reports, Assessment Report (including family health database), IDSR aggregate and line-list database, Health Management Information System (HMIS)/District Health Informative System two (DHIS2) database, Case investigation forms and eDEWS assessment reports within the period of January-December, 2016. Data quality in public health has different definitions from different perspectives. These include: “fit for use in the context of data users, timely and reliable data essential for public health core functions at all levels of government, accurate, reliable, valid, and trusted data in integrated public health informatics networks” [4]. The government of Liberia has been gradually strengthening the national disease surveillance system until the country was hit by the unprecedented outbreak of Ebola virus disease (EVD) in 2014; an epidemic that virtually collapsed the health system. The health system’s weakness was revealed as the result of its inability to detect, investigate and respond to the epidemic in a timely manner; during the outbreak, there was increased deaths and fatality rates among health workers that reflects a weakness in the public health system. The post EVD assessment revealed major weaknesses and the need to establish a national public health institute to focus on building a resilient public health system in the country [1]; ensure preparedness and response; Source of technical expertise in generating, analysing and interpreting public health data in the health sector for the formation of health policies; catalyst for the implementation of international health regulations (IHR, 2005) that are both; sustainable and suitable for the local context; exclusively devoted to overcoming public health challenges and improving the population consciousness on health threats; surveillance, detection, response, and research and generating information to allocate resources for maximal public health benefits. The IDSR system (guidelines) was revitalized in 2015 and 2016 as per lessons learned from the EVD outbreak to serve as a guide for improving early detection, and preparedness activities, improve timely investigation and response, and foster integration to strengthen national cross-sectoral capacity for collaborative disease surveillance and epidemic preparedness, thereby addressing systemic weaknesses within the animal, human and environmental health sectors that hinder effective disease surveillance and response and ensure efficient use of resources [5] The establishment of the national public health institute of Liberia and development of the national investment plan for health resulted in structural reforms-(e.g. the divisions infectious disease and epidemiology (DIDE), division of environmental health were migrated from the MOH and placed under the NPHIL to give examples) in the health sector geared towards a fit for purpose, motivated and productive health work force, re-engineered health infrastructures, and strengthen public health surveillance, epidemic preparedness, diagnostics and response capacity across all levels. The republic of Liberia suffered a devastating civil war which lasted from 1999 - 2003 [6]. The 14 years of civil war destroyed the economy, infrastructures and the health care delivery system such as the hospitals, clinics, electricity, and other essential resources [7, 8]. Liberia was known to be one of countries with the poorest health system in the sub region [4]. As part of the attempts to restore the public health infrastructure of the country, the WHO integrated disease surveillance and response system (IDSR) was adopted in 2004 [9]. The IDSR strategy is an integrated approach for improving public health surveillance and response and promotes the rational use of resources [10]. It is aimed at improving the use of information for early detection of outbreaks and timely response. In the African region, IHR (2005) is implemented in the context of IDSR. The implementation of IDSR in Liberia concentrated on case detection, notification, investigation and confirmation, outbreak preparedness and response, data management and analysis, monitoring and evaluation and support functions (laboratory services, supportive supervision and training based on identified gaps) [11] -prior to the EVD epidemic, the IDSR implementation was limited to AFP surveillance and was not robust in timely detection, investigation, and reporting -( there was no standard data collection and reporting tool at the sub national levels of the health system). In view of this, the timeliness and completeness of the IDSR reports was low, inadequate case detection, reporting, investigation and response to outbreaks, no or inadequate documentation of IDSR data and counties were not using surveillance data for public health actions. It was also observed that data management, and analysis at the sub-national level was very low [12]. The MoH/NPHIL in collaboration with the World Health Organization (WHO) and other partners implemented surveillance activities in all counties and districts, 769 health facilities, and implemented community event-based-surveillance in half of the country to increase for 14 immediately reportable conditions. Over 2,000 health workers were trained in IDSR with at least two officers from each health facility. The early warning and IDSR data is published weekly by the division of infectious disease and epidemiology (DIDE) of the national public health institute of Liberia (NPHIL)/Ministry of Health (MoH). This bulletin highlights numbers of immediately reportable diseases as well as reporting coverage by health facilities, districts and counties during each epidemiologic week. The information is generated from surveillance activities captured by surveillance officers at district and county levels inclusive of laboratory. Over a period of time, the reporting and analysing of IDSR data has improved but the data source remains the county surveillance officers and laboratory only. Efforts are being made in coordination and active involvement of parallel divisions like family health, expanded programme on immunization, and health management evaluation and research at both national and county level to contribute in the collection, correlation and analysing of surveillance data for the priority diseases. In ensuring reliability, quality and credibility of IDSR data generated from the grass root health facilities, the MoH division of infectious disease and epidemiology along with implementing partners commenced the process IDSR data quality audit across the country in selected counties and health facilities. The objective of this paper is to document data quality, identify technical, managerial, and organizational determinants within the Liberian context and inform an operational plan to address data quality needs for IDSR activities.

## Methods

**The data quality audit was conducted in five phases:** phase one focused on the preparatory activities, phase two involved desk review of reported data, phase three activity was field assessment, during phase four, an action plan was developed, and phase five focused on the compilation of data, report writing, and results dissemination to stakeholders. This exercise was carried out from January 2-April 17, 2018 (Table 1).

**Table 1:** Data quality assessment (DQA) activities implementation schedule, 2017

Phase 1: preparation	January 2-March 14, 2017
	Identifying resources, partners, and staff, scheduling activities, venues, and transport, finalizing protocol and data collection tools (printed or electronic). An estimated budget should be developed along with a timeline and agenda of planned activities.
**Phase 2: desk Review**	**January 16-20, 2017**
	Review IDSR surveillance information flow by developing a data flow schematic. Review IDSR surveillance data disaggregated by sub-national levels Select Field Assessment sites. **Timeline: 1 week**
**Phase 3: field assessment**	**March 13-April 1, 2017**
**Includes planning**	**Identify Teams** A 3-day training on assessment protocol and piloting of questionnaires. Conduct assessment and summarize findings. **Timeline: 14 days**
**Phase 4: develop an action plan**	**April 3-4, 2017**
	Develop an action plan for next steps to address challenges, weaknesses, and improvements. Use assessment finding to inform supportive supervision plan. **Timeline: 2 days**
**Phase 5: Report writing**	Analyze data.Develop draft report for inputs from staff and partners. **Timeline: 8 (working) days**
	**April 17, 2017**
**Result dissemination**	Presentation of results/findings. **Timeline: 1 day**

**Figure 1:**
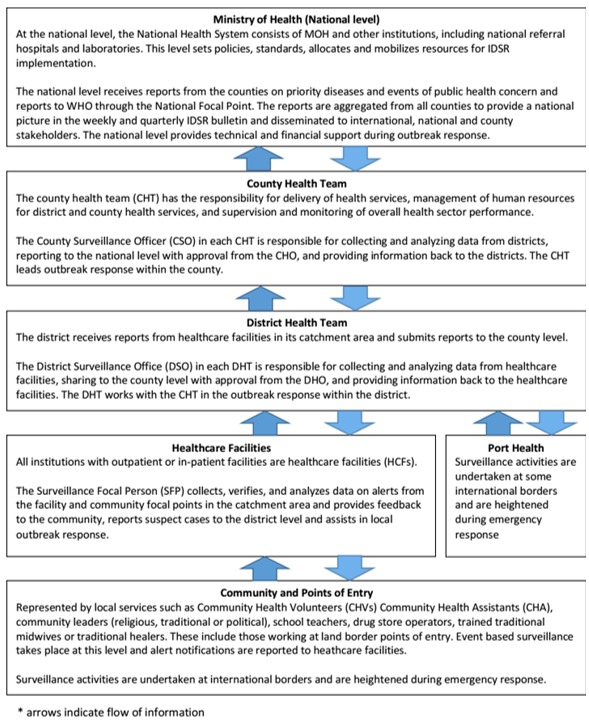
Integrated disease surveillance and response (IDSR) flow of information at each level of Liberia’s public health system

**Sampling Method:** multi-stage cluster sampling that included stage 1: simple random sample (SRS) of five counties, stage 2: SRS of two districts and stage 3: SRS of three health facilities. Pilot assessment was done in Montserrado County with Liberia institute of bio medical research (LIBR) inclusive. A total of thirty (30) facilities was targeted, twenty-nine (29) of the facilities were successfully audited: one hospital, two (2) health centers, twenty-six (26) clinics and respondents included: health facility surveillance focal persons, ownal surveillance officers (ZSOs), district surveillance officers (DSOs) and County surveillance officers (CSOs) (Figure 1).

**Data quality assessment (DQA) tool:** the WHO DQA tool was used for the assessment. The tool was piloted in Montserrado County. Techniques used for data collection were direct observation of data collection and management at all levels. At the health facility, the source of data (patients ledgers and surveillance ledgers) were reviewed, surveillance officers at that level were observed documenting the data or information, interviews and focused group discussions with health facility surveillance focal persons, ZSOs, DSOs and CSOs were conducted by the assessment team form NPHIL/MOH/WHO/ CDC/ John Hopkins University (JHU). Surveillance staff background and training history, competency, specimen collection procedures, case reporting procedures, data analysis, interpretation, and use, support Infrastructure, data Sources, validation of maternal deaths, acute bloody diarrhea, measles, and acute flaccid paralysis, IDSR weekly reporting form, health management information system, health facility registers & charts, identification of key areas for improvement, action items, supervision, mentorship, and feedback and perceptions, knowledge, and attitudes about surveillance.

**Figure 2:**
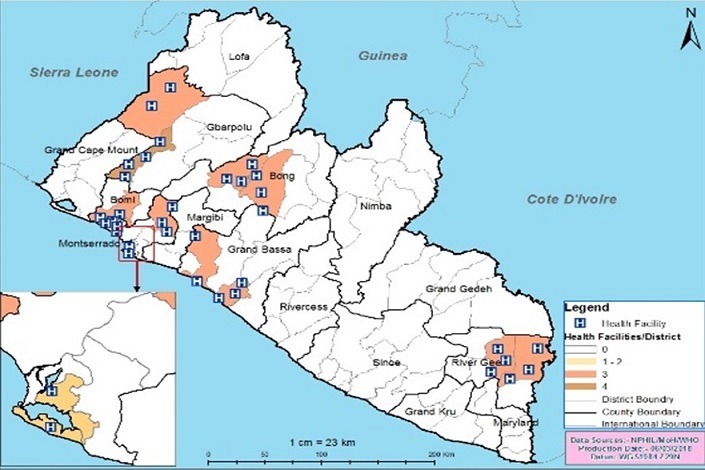
Graphical distribution of study facilities, Liberia, 2017

**Key informants:** these were the surveillance focal persons, district surveillance officers and County ourveillance officers. An interviewer-administered questionnaire was used to interview key informants to assess their knowledge on data quality and operations of the surveillance system. The field assessment was carried out by five (5) teams comprising of four (4) persons. One team was assigned to each county. Each team was made up of a NPHIL staff (National level), a surveillance officer (County level) and a staff from supporting agencies such as WHO, CDC and JHU. Permission was sought from the MoH and NPHIL to carry out this study as part of strengthening public health surveillance in Liberia. Verbal or written consent was obtained from all interviewees and confidentiality was guaranteed, and the questionnaire for this data quality assessment was structured according to the levels of Liberia health care delivery system (Figure 2).

**Figure 3:**
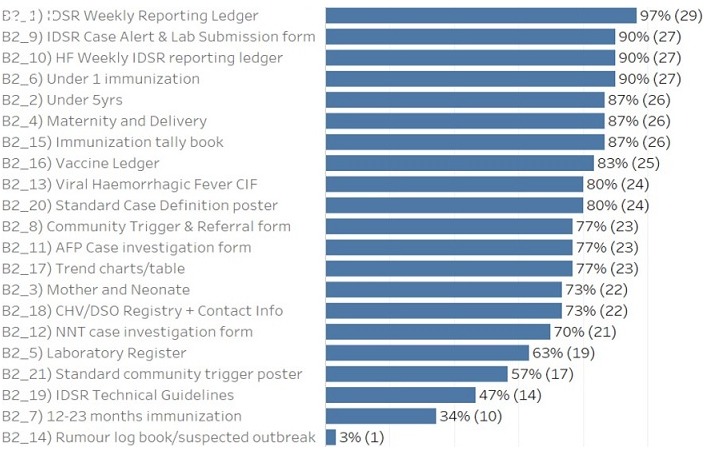
Data sources at health facilities, data quality audit, 2017

**Data quality audit process:** as part of the monitoring and evaluation strategies for continuous improvement in surveillance activities, a data quality audit (DQA) was conducted in five selected counties to assess the quality of data generated and determine factors that influence the quality of surveillance data within the IDSR reporting system. A descriptive cross-sectional study was conducted in 30 health facilities A multi-stage cluster sampling technique was used. Five counties (Montserrado, River Gee, Bong, Grand Bassa, and Gbarpolu), were initially selected using simple random sampling. The districts under each county were classified as urban and rural. In each stratum, one health district was selected giving a total of ten (10) health districts. Within each health district three (3) health facilities were randomly sampled making a total of 30 health facilities (Figure 3).

## Results

**Figure 4:**
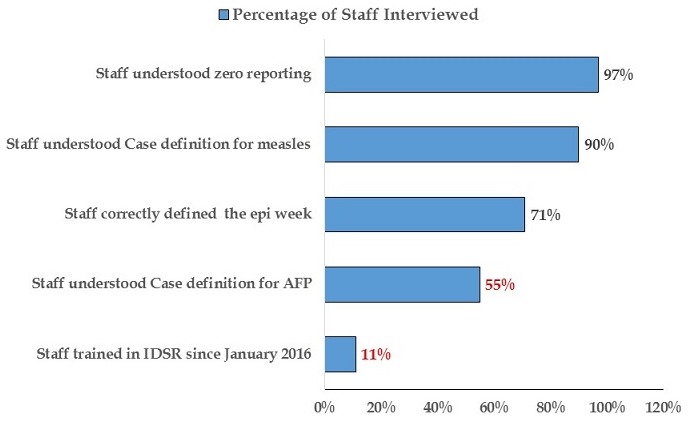
District level staff, data quality audit, Liberia, 2017

**Figure 5:**
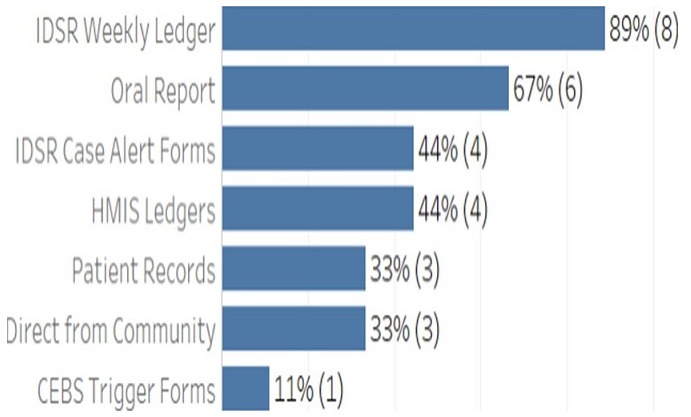
Display of data in health facility for IDSR implementing, Liberia, 2017

The findings indicated that 23% (7/29) reported a dedicated phone for the health facility, 20% (6/29) reported no cell phone network, 17% (5/29) reported daily access to internet, 56.6% (17/29) reported a consistent supply of electricity, no facility reported access to functional laptop and 70% (21/29) reported access to a motorbike for community visits (Figure 4). The findings also indicated that all the DSOs correctly recalled the definition of zero reporting, case definition for measles, and case definition for maternal death. Only one DSO did not correctly recall epidemiology week, and AFP case. Recall of diseases under surveillance was >80% for all conditions (Figure 5). During the data quality audit, it was observed that 40% of health facilities have experienced a stock out of lab packaging supplies in the past year; Stock outs lasted an average of 2 months; All types of packaging was reported as low stock, including red top tubes, purple top tubes; About half of the surveyed health facilities delivered specimens through Riders, about half were assisted by the DSO while there was a large variety in the reported packaging process, with many staff unable to give clear processes. The findings during the exercise indicated that 91% of health facility staff were mentored on data quality check and data management including the importance of the timeliness and completeness of reporting through supportive supervision and mentorship; 65% of the health facility assessed received supervision on IDSR Core Performance Indicator; and 58% of the health facility Officer In Charge gave feedback to the community level. It also indicated that, 78% of the health facility use Bar chart as methods to detect outbreaks; 56% used trend lines; 33% used summary table and 11% used map to determine outbreaks. The findings further indicated that for routine data harmonization at the peripheral level of the IDSR implementation, 90% of the DSOs routinely check the DHIS2 platform for data consistency with IDSR data reported the same period; it was also observed that 40% of the health facility focal persons lack training in data management; while 85% of the health facility recorded information on cases detected at community level.

**For the data analysis and use:** a checklist as per WHO guidelines for data quality audit was used to assess key indicators of quality surveillance data. A vicariate and bivariate analysis was conducted to summarize data collected using Epi Inform™ version Most health facilities reported using data to inform topics during the health talks. 100% of the DSOs assessed, correctly recalled the definition of zero reporting, case definition for measles, and case definition for maternal death and high numbers of disease are used for sensitization, specifically around measles and diarrhea 1 DSO did not correctly recall the epidemiologic week, and AFP case. Recall of diseases under surveillance was >80% for all conditions.

## Discussion

Sound and reliable data quality assessment is vital to obtain the high data quality which enhances users’ confidence in public health and their performance. As Liberia monitors and evaluates the performance and progress of IDSR indicators, the need for data quality assessment in public health information system that store the performance-and-progress-related data needs to be routinely undertaken to ensure generation of credible, reliable and quality data. High quality data and effective data quality assessment are required for accurately evaluating the impact of public health interventions and measuring public health outcomes. Data use, and data collection process as the major dimensions of data quality, all need to be continuously assessed for overall data quality. Data quality audit “DQA” has been routinely conducted by MoH/NPHIL-Liberia as part of the IDSR implementation strategy to improve disease surveillance and the information generated helps to improve training, supervision, and reporting tools of the program across the country. Data are essential to public health. They represent and reflect public health practice. The broad application of data in IDSR for the evaluation of public health accountability and performance has raised the awareness of NPHIL, MoH, WHO and public health agencies of data quality, and of methods and approaches for its assessment [13].

We systematically reviewed the current status of quality assessment for each of the three dimensions of data quality: data, data collection process and data use. The results suggest existence of data capture tools at all levels of health care system with most assessed indicators above 80%. Our findings based on the proposed conceptual framework of data quality assessment for public health identified gaps in reporting aggregated IDSR data not harmonised with DHIS2, and limited data use for decision making. Data quality is influenced by technical, organizational, behavioural and environmental factors. It covers large information systems contexts, specific knowledge and multi-disciplinary techniques. Data quality audit is frequently done as a component of the quality or effectiveness or performance of the IDSR. However, data quality assessment hidden within other scopes may lead to ignorance of data management and thereby the unawareness of data quality problems enduring in public health practice. Data quality needs to be positioned at the forefront of IDSR as a distinct area that deserves specific scientific research and management investment.

While this review provides a detailed overview of data quality assessment issues, there are some limitations in its coverage, constrained by the access to the databases and the breadth of public health information systems making it challenging to conduct systematic comparison among studies. The importance of systematic, scientific data quality assessment needs to be highlighted. All three dimensions of data quality, data use and data collection process, need to be systematically evaluated. The quality of data use and data collection process has not received adequate attention. This lack of recognition of data use and data collection process might reflect a lack of consensus on the dimensions of data quality. Further development in methods to assess data collection process and data use is required. Effort should also be directed towards clear conceptualisation of the definitions of the relevant terms that are commonly used to describe and measure data quality, such as the dimensions and attributes of data quality. Data quality assessment was mixed methods (qualitative and quantitative assessment methods) to assess data from multiple sources (records, organisational documentation, and data collection process and data users) and used at health facility, district and county levels. The validity of a study would be doubtful if the quality of data could not be verified in the field, especially when the data from data capture tools are varied with data submitted to national level has no errors. This was limited by the coverage to 5 out of 15 counties and to the databases of IDSR, DHIS2 and data capture tools at health facility, district and county levels. Further research could develop consistent data quality definitions, attributes, quality of data use and the quality of data collection process. Data-use and data-process have not been given adequate attention at health facility, district and county levels, although they were equally important factors which determine the quality of data.

**Recommendations:** conduct regular data harmonization/audit at subnational level to ensure that health workers are knowledgeable on the importance of quality and reliable data. Capacity building for health workers at sub national level in data management. Ensure the institutionalize data management training in pre-service and academic institutions, on-going in-service refresher trainings in data analysis for public health actions. Establish an effective system to improve data harmonization at the subnational level.

## Conclusion

Public health is a data-intensive field which needs high-quality data and authoritative information to support public health assessment, decision-making and to assure the health of communities. Data quality assessment is important for public health. In this review Completeness, accuracy, and timeliness were the three most-assessed attributes. Quantitative data quality assessment primarily used descriptive surveys and data audits, while qualitative data quality assessment methods include primarily interview, questionnaire administration, documentation review and field observation. We found that data-use and data-process have not been given adequate attention, although they were equally important factors which determine the quality of data. Other limitations of the previous studies were inconsistency in the definition of the attributes of data quality, failure to address data users’ concerns and a lack of triangulation of mixed methods for data quality assessment. The reliability and validity of the data quality assessment were rarely reported. These gaps suggest that in the future, data quality assessment for public health needs to consider equally the three dimensions of data quality, data, data use and data process. Measuring the perceptions of end users or consumers towards data quality will enrich our understanding of data quality issues Data use is limited to risk communication and sensitization, no examples of use of data for prioritization or decision making.

### What is known about this topic

- Data quality audits lead to improvement in over reporting and under reporting which compromise informed decisions, integrity of generated data, quality assurance standards and compliance levels;
- Public health is a data-intensive field which requires high-quality data to support public health decision-making;
- Improving data quality requires good data capturing tools that are capable of analysing the quality of data generated based on data quality audits.

### What this study adds

- Forty percent (40%) of surveillance officers at health facility level lack training in data management;
- Undefined data management strategy and quality level makes data generated overwhelming and less useful in decision making;
- Regular data quality audit is necessary to understand the actual status of data quality and integrity issues in the health care delivery system and addresses data quality challenges aimed at improving data consistency, reliability and informed decisions.

## Competing interests

The authors declare no competing interest.
